# Investigating Italian Consumer Preferences for Different Characteristics of Provolone Valpadana Using the Conjoint Analysis Approach

**DOI:** 10.3390/foods9121730

**Published:** 2020-11-25

**Authors:** Niculina Iudita Sampalean, Tiziana de-Magistris, Daniele Rama

**Affiliations:** 1 Department of Agri-Food Economics, Faculty of Agriculture, Food and Environmental Sciences, Campus Piacenza-Cremona, Catholic University of Sacred Heart, via Emilia Parmense, 84, 29122 Piacenza (PC), Italy; niculinaiudita.sampalean@unicatt.it (N.I.S.); daniele.rama@unicatt.it (D.R.); 2 Unidad de Economía Agroalimentaria y de los Recursos Naturales, Centro de Investigación y Tecnología, Agroalimentaria de Aragón, 50059 Zaragoza, Spain; 3 Instituto Agroalimentario de Aragón (IA2), CITA-Universidad de Zaragoza, 50013 Zaragoza, Spain

**Keywords:** Provolone Valpadana, cheese, conjoint analysis, cluster analysis, market shares

## Abstract

The objective of this paper was twofold. First, we estimated consumer preferences for an Italian cheese (Provolone Valpadana) with respect to several attributes and levels, such as price, origin certification, production system, ‘free from’ labelling, and brand. Second, we identified consumer clusters with similar preferences for various cheese characteristics. Preferences were estimated using the conjoint analysis method. Then, a cluster analysis was used to classify consumers into different (three) clusters followed by a market simulation. In all three clusters, the attribute most preferred by Italian consumers was the brand of the cheese: consumers preferred to purchase Provolone cheese having the lowest price, produced by Auricchio, bearing a European Union (EU) quality certification, produced organically, and non-lactose-free. The results of our study provide helpful information to food companies for better segmenting their market and targeting their consumers, as well as effectively promoting their products using brands, certifications as organic and lactose-free. This study contributes to the literature on consumer preference for the EU labelling scheme (voluntary and mandatory). To our knowledge, this is the first study to investigate this combination of multiple labels displayed on the front of Italian cheese packaging.

## 1. Introduction

Consumers have become highly conscious of food product characteristics when shopping. They search for products of higher quality made with environmentally friendly production processes that have connections with the territory and that prevent health issues [1,2,3,4]. To keep up with this change in consumer preferences, a greater range of products have emerged on the market. As a result, this abundant variety of products has made food shopping a more difficult task for consumers [5].

Some food-labelling schemes enable manufacturers to disclose the qualities of food products to consumers [6]. Thus, the use of labels allows food companies to signal quality or the presence of specific desirable but not obvious attributes [7]. In this context, during the past two decades, the European Union (EU) has launched different food labelling regulations that food companies can adopt on a voluntary basis. The aim of these food-labelling schemes is to establish the requirements for the use of particular labels and the procedures for controlling the quality of labelled products to protect consumers from being misled. Two examples of these regulations are EEC Regulation No. 834/2007, concerning organic production and the labelling of organic products, and Regulation No. 2081/92 adopted by the European Commission and further amended with several texts which culminated in EEC Regulation No. 1151/2012, which introduced the quality labels Protected Designation of Origin (PDO) and Protected Geographical Indication (PGI) labels. The PDO and PGI labels were designed as quality cues to reduce asymmetric information and to reduce consumer uncertainty associated with food purchases regarding desirable product characteristics. In particular, the PDO label may be used in the case of food products that are produced, processed, and prepared in a given geographical area using local know-how. (EEC Regulation No. 1151/2012).

Many studies on consumer preferences for food labelling have been conducted since the 2000s, and they have especially focused on consumer willingness to pay (WTP) for multiple front-of-package (FOP) food labelling schemes [1,3,8,9,10] and some have focused on cheese products [11,12,13].

Although cheese is one of the most important agricultural food products in Italy, accounting for 87.4% of the total value of Italian dairy products sold abroad [14], studies on consumer preferences for food labelling on cheese products are scarce [12,15,16,17]. In general, these studies have reported that PDO labels are preferred over organic labelling; nutritional claims are preferred over organic labelling; nutritional claims are preferred over the nutritional fact panel. Yet, most studies have also demonstrated that preferences for food labelling schemes are heterogeneous across consumers, with gender, age, educational level, and that the degree of environmental concern influenced consumer WTP for different cheeses [11,18].

At the European level, 53 of 247 types of cheese designated as PDO or PGI are Italian produced. For Italy, these cheeses represent 57% of the national production value and 50% of exports. Likewise, in 2018, sales of Geographical Indication (GI) cheeses grew by 2% in quantity and 1.5% in value compared with non-GI cheeses; those cheeses decreased by 1.6% in volume and increased by 0.6% in value [19]. In this context, Grana Padano PDO and Parmigiano Reggiano PDO together account for 59.3% of the volume and 66.3% of the value of total sales of GI cheeses in large-scale distribution [19].

Four regions of northern Italy (Lombardy included) account for 65% of the PGI production value; 76 PDO/PGI certified products come from Lombardy, and 9896 PDO/PGI enterprises are located in the region, generating a production value of €1958 million [19].

Provolone Valpadana is one of the most consumed GI cheeses in Italy, with a consumption value of €77 million and representing 1.1%, by weight, of the total Italian GI cheese consumption, more than the well-known Pecorino Toscano PDO or Montasio PDO [19].

Istat’s ‘Report on Family Consumption Expense’ shows that the average monthly household expenditure in Italy in 2017 was €2563.94. Of this total average expenditure, €457.12 was dedicated to food and non-alcoholic drinks. Spending on cheese, milk, and eggs was €58.26 (these are measured as an aggregate), which represents 2.27% of the total average monthly household expenditure and 12.75% of the total average monthly expenditure for food and non-alcoholic drinks. Provolone is a spun-paste semihard cheese made from whole cows’ milk with natural acidity from fermentation. Milk is collected in the area of origin within 60 h of milking and then undergoes mild heat treatment until the cheese is pasteurised. The maturation period can vary as follows: for up to 6 kg, the minimum maturation period is 10 days; for over 6 kg, the minimum maturation period is 30 days; for over 15 kg of only the piquant variety, the minimum maturation period is 90 days; for over 30 kg of the product labelled PVS (Provolone Valpadana Strong) the maturation period is over 8 months (for the piquant variety). The cheese may also be smoked, formed into different shapes (one of its most important characteristics) and be formed into blocks of various weight [20].

Provolone Valpadana was recognized as a PDO product in 1996. Since then, all its producers must strictly comply with the production regulations. Today, Provolone Valpadana PDO is produced by authorised cheesemakers and can also be sold by packaging companies. In all cases, every single shape must be marked with the Consortium’s trademark and the PDO trademark, which are unequivocal guarantees of quality [21].

Provolone Valpadana is a typical premium cheese with unmistakable qualities; it is easy to compare with other generic products (e.g., spun-paste cheese), but consumers must recognise its unique qualities.

In light of the foregoing context, the objective of this study was twofold. The first objective was to assess Italian consumer preferences for Provolone Valpadana that bears multiple food labels, such as those making claims related to their origin (PDO), method of production (organic), and healthiness (free from lactose). Because of the increasing complexity of consumer preference, the second aim of this study was to examine heterogeneity in preferences for labelled cheeses on the basis of consumers’ sociodemographic and personal characteristics.

To achieve these objectives, we conducted an online survey of 246 Italian cheese consumers and used both a conjoint and cluster analysis to analyse our data.

Our study contributes to the consumer cheese preference literature because this is the first study to investigate consumer preferences for these multiple food labels on Italian cheese using an approach combining conjoint analysis and cluster analysis. Furthermore, our findings have marketing implications because they disclose useful information to Italian stakeholders along with the cheese supply chain that can help producers and retailers, among others, design the best strategies to differentiate cheeses by adopting various labelling schemes for different segments of consumers.

### Literature Review

Consumer preferences towards different food labels and different combinations of food labels have been widely investigated during the last decade for several food products. For instance, Bernabeu et al. [22]. analysed the price, origin, type and production system for wine, while Erraach et al. [9] examined the relative importance of European origin labels (PDO) associated with extrinsic (price and packaging) and intrinsic (color) cues of Spanish olive oil on consumer preferences. Aprile et al. [8] provided evidence about extra-virgin olive oil consumers’ WTP. Likewise, de Magistris and Gracia [23] investigated consumers’ preferences and WTP for almonds carrying different sustainability labels (e.g., food miles and organic certifications). Similarly, Mesias et al. [24] used price, origin, production system and labelling as attributes to study preferences for beef in Extremadura. Finally, Cilla et al. [25] and Garavaglia and Mariani [26] investigated consumers’ preferences for cured ham in Spain.

These studies in general showed that the PDO and organic farming labels are most important. In particular, Aprile et al. [8] showed that the highest premium price is associated with PDO labels, followed by organic farming labels, and then PGI labels. Erraach et al. [9] indicated that price and origin labelling (PDO label) were the attributes that most affect consumers’ preferences. De Magistris and Gracia [23] suggested that consumers were willing to pay a positive price premium for ‘locally grown’ and ‘organically produced’ attributes. Finally, Mesias et al. [24] showed that the origin of the product is the most important attribute in the choice of beef, followed by quality labelling, production system and price. In the literature, some studies have focused on consumer preferences for several food labels appearing on cheeses sold in Europe. For example, Garavaglia and Marcoz [12] investigated the Fontina cheese bearing the PDO certification. The authors reported differences in consumer preferences depending on the respondent’s place of residence. Likewise, Skubic et al. [13] conducted a study in Slovenia and found that price was the most important attribute for consumers. De Magistris and Gracia [27] found that Spanish consumers were willing to pay more for PDO cheese than for organic or light cheeses. Moreover, gender, age, educational level, and the degree of the environmental concern influenced consumer WTP for different cheeses. 

Several studies have investigated consumer preferences for cheese products based on production, processing, trading or branding. For example, Tendero and Bernabeu [28] demonstrated that consumers most highly value the PDO label as a guarantee of quality and food safety and that they prefer cheeses that are appropriately priced, aged, and if possible, certified. Similarly, Bernabeu et al. [29] investigated the perceived quality of cheese from Castilla-La-Mancha compared with that of cheeses from the rest of Spain and foreign cheeses. The authors found that consumers attached the most importance to the origin of the cheese, followed by the cheese type, price, and production system (organic). Moreover, Bernabeu et al. [29] revealed that organic production systems had the lowest relative importance as well as a negative influence on consumer preferences for cheese.

Napolitano et al. [17] assessed the effect of information concerning organic production of Pecorino cheese on consumer WTP. They showed that consumers were willing to pay a higher price than local retail price for organic cheese than regular cheese (€3.00/100 g). Moreover, information regarding organic farming was found to be the main determinant of cheese preference and consumer WTP.

Some studies have focused on consumer WTP for cheeses with FOP nutritional claims. For instance, Gracia and de Magistris [3] determined that the most preferred food label was the PDO indication, closely followed by the nutritional fact panel and the EU organic logo. Likewise, de Magistris and Lopez-Galan [11]. Investigated consumer WTP for cheeses bearing ‘reduced fat’ and ‘low salt’ claims in Spain. The authors reported that Spanish cheese consumers were willing to pay a premium for packages of cheese with reduced-fat and low-salt claims.

Another important claim related to the health of consumers that may be present on the FOP of cheese is the ‘lactose-free’ claim. This claim contains important information for consumers with food intolerances (e.g., lactose intolerance). Hartmann et al. [30] investigated how ‘free-from labelling’ shapes consumer perceptions of food products and whether the absence of an ingredient is considered an indicator of improved nutritional value of the product. The authors concluded that a ‘lactose-free’ label was highly correlated with the perception of healthiness as well as the intention to pay a premium price, imagining that consumers attribute inappropriate health benefits to products free of lactose.

Lactose-free cheese occurs when cheese is aged more than about one month, as bacteria added from the beginning to the milk ‘digest’ lactose converting it into lactic acid; the latter contributes to cheese taste and aroma, and first of all it induces protein coagulation, therefore the cheese production [31].

Finally, studies on consumer preference for private brands of cheese are scarce. To our knowledge, only Arfini [15] conducted such a study, comparing consumer WTP for cheeses bearing Consortium labels and EU PDO labels. The findings showed that the presence of the manufacturer’s brand was comparatively important when shopping for grated cheese (18.91%) and ordinary cheese (18.24%). The results further indicated that little attention was paid to the private label of the firm producing or marketing the typical products at issue; however, the Consortium label played an important role in reassuring consumers about the quality of the purchased product. For Parmigiano Reggiano cheese, as many as 75% of the interviewed consumers looked for the Consortium label when shopping [15].

## 2. Materials and Methods

### 2.1. Conjoint Analysis and Choice Task Procedure

Conjoint analysis is one of the most widely used marketing research methods for analysing consumer trade-offs and is used to examine survey responses concerning preferences and intentions to buy; it is a method for simulating how consumers might react to changes in current products or new products introduced into an existent competitive array [32]. The model assumes that the substitute products notion can be defined as a series of specific levels of a common set of attributes. It also assumes that the total utility the consumer derives from a product is determined by the utility or part-worth contributed by each attribute level. The conjoint analysis starts with the consumer’s overall or global judgements about a set of complex attributes. It then performs a decomposition of the original evaluations of the consumer into separate and compatible utility scales, according to which the original overall judgements can be reconstituted [33]. 

The most commonly used composition rule adopted in this study is additive because it is the one considering most (80% or 90%) of the variation in preference in almost all cases. Following Mesias et al., 2005 [24] we used the additive composition rule:

$$Y=\overset{n}{\underset{i=1}{\sum }}X_{\mathrm{ij}}\underset{j=1}{\sum }v_{\mathrm{ij}}$$

where Y is the total utility, Vij is the utility associated with level j (j = 1, 2 …, m) of attribute i (i = 1, 2 …, n), and Xij is a dummy variable that takes the value of 1 (or 0) in the case of presence (or absence) of the jth level of the ith attribute.

For the qualitative attributes, the part-worth model was used because of its flexibility [31]. For the price attribute, a Linear Less relationship was used because in general, higher prices correspond to lower utility or preferences.

Here, the conjoint analysis (CA) procedure consisted of a choice task to establish the trade-offs that Italian consumers make between price, quality certification (PDO), system production (organic), ‘free from’ labelling, and brand of Provolone Valpadana Dolce. 

Italian consumers were presented 16 varieties of Provolone Valpadana Dolce with different combinations of attributes levels. The attributes (and their levels) used in the study are listed in Table 1 and were identified (as most important when buying cheese: price (€2.20/200 g, €2.86/200 g, and €3.20/200 g), quality certification (with or without PDO), production system (organic or conventional), brand (Auricchio, Latteria Soresina (both national brands), Coop (private label), or no brand) and lactose content (lactose-free or non-lactose-free). To select the price levels, information on the Provolone Valpadana sold in different Italian supermarkets was used, and three price levels were set up (€2.20/200 g, €2.86/200 g, and €3.20/200 g). The most frequent lowest price of a package of 200 g was around €2.20. Therefore, this price was chosen as the lowest price. The second level was 30% higher than the most frequent lowest price, and the third level was 45% higher than the most frequent lowest price. Moreover, the Coop private label was chosen since it could represent any Italian private label of Provolone Valpadana. As for the national brands, two brands were selected: Auricchio, the leader brand [34], and Latteria Soresina, another national brand of Provolone Valpadana cheese. In other words, any other private label and national brand could have been used instead of COOP and Latteria Soresina.

With these five attributes and their 13 levels, 96 potential profiles were obtained. Because this is a particularly high number to show to consumers, an orthogonal design (SPSS) was applied to the profiles to reduce the number to 16, a number more likely to encourage participation of consumers.

**Figure 1:**
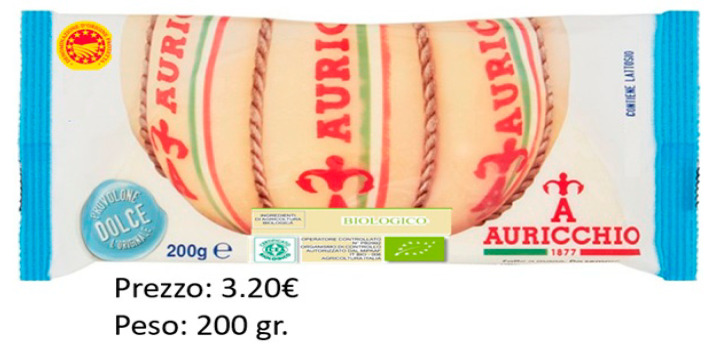
Example of evaluated cheese profile.

An example of one of the 16 obtained profiles is shown in Figure 1.

The respondents evaluated a picture that contained the different attribute levels in this study. The packaging used was the same as that on the market, but some attribute-level combinations shown are not present in the marketplace. Once the profile’s cards were formed, they were shown to the respondents who evaluated them, giving a score from 1 to 10 according to their stated preference. Participants had the option to repeat the same number on different cards. The number 1 and 10 corresponded to the lowest and highest levels of preference, respectively (thus employing the complete profile method). The main argument in support of the full-profile approach is that it gives a more realistic description of stimuli by defining the levels of each of the attributes and considering the potential environmental correlation between factors in real stimuli [35].

Using SPSS software, the utility estimates generated by the conjoint analysis were used in a k-means cluster analysis to classify the consumers into homogeneous preferences groups and classify them into clusters.

### 2.2. Data Gathering

A self-administered structured electronic survey was completed by 245 people in Italy. The survey was distributed online (Facebook, Messenger, WhatsApp, LinkedIn) between September 2019 and March 2020. All subjects gave their informed consent for inclusion before they participated in the study. The study was conducted in accordance with the Declaration of Helsinki, and the protocol was approved by the Ethics Committee of Catholic’s University of the Sacred Heart. A pilot study with Valpadana cheese consumers was conducted to test the validity and the comprehension of the survey. Table 2 shows the characteristics of the final sample of respondents.

Approximately 81% of the respondents were women and only 19% were men. Compared with the Italian population, women were overrepresented, which might be explained by the fact that, in general, women oversee food shopping for households. Roughly 24% of the participants were less than 35 years old, 53% were between 36 and 55 years old, and 23% were older than 55 years. The sample may have skewed toward a younger demographic because the questionnaire was distributed online, and the older population tends to not have access to the Internet or computer skills. Roughly 43% of the participants had completed high school degrees, and 57% had a university degree. More than half were married (53%), which is similar to the Italian population overall (47%). In more than 40% of our sample, the family was composed of three or four members, which is also comparable with the Italian population overall (35%). More than half the respondents (66%) were from southern or island regions, with the central region being the least represented territory (7%) of participants compared with 20% of the Italian population. For participant occupations, most (40%) were office workers, followed by housewives (14%), freelancers (13%), students or unemployed persons (present in the same proportion of 5%), teachers (2%), and entrepreneurs (2%). Concerning annual income, 26% of the respondents earned less than €10,000 per year, 31% earned between €10,000 and €20,000 per year, and 11% earned more than €40,000 per year.

## 3. Results

The results section is divided into two parts. The first part describes the results of consumer preferences for different Provolone Valpadana cheese profiles and the clusters formed by these preferences. The second part, using the simulation process, identifies market shares for the most preferred cheeses as well as for some existent cheeses on the market.

### 3.1. Cheese Consumer Preference Structure

Table 3 presents consumer preferences for various FOP food labelling. We found that brand is the most highly valued attribute (58.24%), followed by price (13.89%), production system (10.65%), lactose content (8.94%), and finally, the quality certification (8.29%). Additionally, based on the utility estimates for each level of attributes, consumers preferred to purchase Provolone cheese having the lowest price, bearing the Auricchio brand, bearing the EU quality certification, having been organically produced, and non-lactose-free.

Using SPSS software, the utility estimates generated by the conjoint analysis were used in a k-means cluster analysis to classify the consumers into homogeneous preferences groups and classify them into clusters. Three clusters of consumers were identified with different preference structures. 

The analysis of variance (ANOVA) showed that all clusters differed significantly from each other with respect to price (*p* < 0.05), lactose content (*p* < 0.1) as well as for the other variables were the significance was the highest (*p* < 0.01).

The utility estimates and their relative importance were then calculated for each of the levels of the attribute. The results, together with the size of each cluster (expressed as %), are listed in Table 4 (A and B).

In all three clusters, the attribute most preferred by the Italian consumers was the brand of the cheese, and this attribute’s highest relative importance was in cluster 2 (77.16%) followed by cluster 1 (62.83%).

Regarding the largest and first cluster (44%), the most preferred attribute was the brand (62.83%), followed by price (13.57%). However, these consumers equally preferred the production system and lactose content (8.79% and 8.38%), whereas the quality certification was the least preferred attribute (6.43%).

The second cluster consisted of 22% of the sample who most preferred the brand of cheese (77%); lactose content and price exhibited similar relative importance of preference (6.52% and 6.49%, respectively). As in cluster 1, EU quality certification was the least valued attribute (4.53%). Comparable to the first and the second cluster, in the third cluster (representing 34% of all consumers), the most preferred attribute was brand, followed by price (19.13%); however, in contrast to the other two clusters, the lactose content (11.23%) was the least preferred attribute.

Finally, as expected, the level of price in all clusters exhibited a negative value, especially for the highest price (€3.20/200 g). Moreover, the utility assigned to the ‘free from lactose’ level exhibited a negative value in all clusters, suggesting that consumers did not prefer lactose-free cheese.

Consumers in cluster 1 showed a clear preference for cheese without a quality certification, in contrast to clusters 2 and 3. Likewise, regarding the brand attribute, cheese with the Auricchio brand presented the highest utility in the first and second clusters but negative utility in the third cluster, in contrast with Latteria Soresina and Coop, both of which exhibited positive utility. Thus, the results suggest that consumers in cluster 1 most preferred the brand Auricchio while in the cluster 3, consumers most preferred the cheese brand Coop.

Regarding lactose content, in cluster 2, consumers ranked the lactose content in the cheese as the second most important attribute in contrast to consumers in clusters 1 and 3. As for the production system, except for cluster 1, all consumers in clusters 2 and 3 ascribed a positive value to the EU quality certification.

### 3.2. Cheese Consumer Profiles

To better define consumers from each cluster, consumer profiles were interpreted by looking at their socioeconomic characteristics (Table 5).

Table 5 shows the Pearson chi-square results of the three clusters and the consumer characteristics that were found to be statistically significantly different across clusters at a 5% significance level (at least). Results indicated that all clusters included a higher proportion of women with a university degree and living in the south or island regions. Although in all three clusters the most predominant gender was female, in cluster 3, we found the highest percentage of men (28%). Cluster 1 consisted of the largest percentage of people aged between 35 and 55 years, and in contrast to clusters 2 and 3, the lowest percentage of older people (>55 years old). Further, 85% were women with high school degrees (49%) or a university degree (51%). Finally, 70% of the cluster consisted of people who lived in the southern or island regions of Italy and living in a family with three or four family members. Cluster 2 consisted of the largest percentage of women between 35 and 55 years of age and, in contrast to clusters 1 and 3, of the lowest percentage of young people (<35 years old). As in the first cluster, 49% of sample had university degrees, and 83% lived in the southern or island regions in families of three or four members. In cluster 3, 35% of the individuals were between the ages of 18 and 35, 36% between the ages of 36 and 55, with a university degree (70%) and living in a family composed of more than four family members. In contrast with clusters 1 and 2, cluster 3 had the highest percentage of men (28%) and people living in the southern and island regions (53%). If we compare our results with the ones found in the literature for different food groups, we see that the price was one of the most important attributes, which is similar to our results. In contrast to our results, the origin was also one of the most valued attributes. For example, Bernabeu et al. [22] identified three consumer segments: one that shop for wine mainly by price, one by origin, and another one by a combination of price and type of wine. Erraach et al. [9] identified four consumer segments, two of which involve liking the origin label. De Magistris and Gracia [23] suggested that consumers’ preferences for locally grown and organically produced almonds were heterogeneous, with three consumer segments identified: ‘conventional consumers’, ‘short distance consumers’, and ‘sustainable consumers’. Through cluster analysis, Mesias et al. [24] identified three groups of consumers. One that gave least importance to a product’s origin and thus obviously valued less whether beef was produced in Extremadura or in other areas. The other clusters gave more importance to the origin, and with one of these clusters, 62% of the weight of the purchasing decision was based on the origin of the product.

### 3.3. Simulation of Market Shares

The results of the conjoint analysis were utilised to simulate choices among the ideal cheese products for each cluster and three other existent Provolone cheese profiles. This analysis was performed using the part-worth utility function for each respondent. The simulation summary gives the probabilities of choosing a particular simulation profile as the most preferred profile under three different probability-of-choice models. The maximum utility model represents the probability of choosing a profile as the most preferred. The BTL (Bradley–Terry–Luce) model computes the probability of choosing a profile as the most preferred by dividing the profile’s utility by the sum of the total utility of the simulation. The logit model is like the BTL model but uses the natural log of the utilities instead of the utilities themselves [28].

Results from the simulation of market shares are shown in Table 6. 

The largest market share corresponds to the ideal product (cheese number 1) for both the study’s total sample and cluster 2, and this share fluctuates between 29.8% and 19.01%. Cheese number 5, an existent cheese, is next, with a market share that fluctuates between 26.1% and 17.2%, and cheese number 2 follows, with a variable market share between 18.5 % and 23.4%, depending on the method used for estimation. Between the first and the second cheese, there was a decrease in market share between 0.5% and 9.9%, depending on the simulation method employed; the decrease was presume it was because the EU quality certification was missing. Thus, adding the quality certification to an existent cheese profile could increase its market share by up to 9.9%. If we look at cheese profiles number 3 and number 4, we can see that the use of the organic production system could increase the market share from 0.7% to 7.2%, building on the distinctive estimating methods.

## 4. Discussion

The first objective of this study was to estimate consumer preference for Italian cheeses with various levels of different attributes such as price, origin certification (PDO), production system (conventional or organic), ‘free from’ labels (lactose-free), and brand (national brands/private labels) on Italian consumer preferences for cheese.

Based on the analysis of these attributes, the results showed that cheese is a highly differentiated food product. Cheese preferences were most positively affected by being produced by a national brand followed by private label brand production, being produced organically, and non-lactose-free. 

Examination of the relative importance of the various attributes indicated that brand was the most important attribute followed by price in two of the three clusters. This result is in contrast to the results found by Skubic et al. [13] who showed that price was the most important attribute for cheese. There were different price levels used in the two studies, and Skubic’s study had four levels, one more than the other. This might have an influence on the final results, but a bigger influence on the price effect could be the set of attributes that were used, considering that the brand (which has the highest importance in our study) is completely missing from Skubic’s study. Our findings also suggest a positive preference for the organic label. This result is in agreement with Napolitano et al. [17] who found that ‘organic’ can be a potential tool for cheese differentiation, particularly for small scale and traditional farms. However, our results are in contrast with those of Bernabeu et al. [29], who suggested that the organic attribute is an inadequate differentiation strategy in the cheese market.

Similar to Arfini [15] our results indicated that national brand was the most preferred cheese attribute when analysing different market clusters.

In contrast to Hartmann et al. [30], we found that consumers did not prefer to buy lactose-free cheese, we found that, in all three clusters, Italian consumers preferred to buy non-lactose-free Provolone cheese.

Additionally, the EU quality certification had a positive impact on cheese preference, except for cluster 1, where consumers preferred noncertified to certified cheese. As for the price, for two out of the three clusters, it was the second most important attribute. These results accord with Tendero et al. [23] who discovered that regular cheese consumers value the cheese having the EU quality guarantee but also ascribe much importance to price. Similarly, Garavaglia and Marcoz [12], studying the value of PDO certification of Fontina cheese and its impact on consumer preferences and WTP, found that the PDO certification has substantial importance on consumer purchasing decisions, persuading them to pay a premium price for products with PDO.

Our findings indicated that consumers value the organic production system more than the PDO certification. This result contrasts with other studies [1,3,8,36,37] that reported that consumers value PDO more than organic certification.

Finally, with regards to consumer heterogeneity, our results coincide with de Magistris and Gracia [27], Krystallis and Ness [38] and Fotopoulos and Krystallis [39], which all found that consumer age, education, and income level are important factors when it comes to selecting food products. In particular, our findings demonstrated significant differences between respondents in terms of geographical distribution, area of origin, sex, age, and education. Similar results were found by de Magistris and Gracia [27], which revealed that female respondents, older respondents, and those with a university education were willing to pay more for quality-labelled cheeses.

## 5. Conclusions

We analysed consumer preference for Italian cheese products with respect to FOP origin certification (PDO), production system (conventional or organic), ‘free from’ labels (lactose-free), price, and brand (national brands/private labels brands), and we explored heterogeneity of consumers. In general, the results indicate that in all three clusters, the attribute most preferred by Italian consumers is the brand of cheese. Italian consumers prefer to purchase Provolone cheese at the lowest price, that carries the Auricchio brand marking, that is EU quality certified, that is organically produced, and which is non-lactose-free. It is expected that most consumers that dislike the ‘lactose-free’ claim are conscious that Provolone is naturally lactose-free (as they are Provolone Valpadana cheese consumers) and consider any manipulation connected to the ‘lactose-free’ claim as a possible modification of the typical taste of the cheese.

The identification of certain segments of the Italian Provolone cheese market could help cheese producers better understand consumer needs and their most preferred attributes; consequently, producers could adapt or diversify their marketing mix, especially in the areas of new products development and communication strategy, in order to better match consumer needs. For example, the best way to attract consumers in the first cluster to buy Provolone Valpadana cheese is to use an organically labelled national brand at a fair price. Moreover, because this cluster consists of those people who are married and with children, the communication strategy should accentuate the benefits of organic products on health, especially for children and older individuals.

In the second cluster, people most preferred the national brand and cheese with lactose, and thus, producers should highlight the uniqueness of the flavour, appearance, and texture of their Provolone cheese to indicate how lactose content affects these characteristics. Also, they should emphasize the enjoyment of Provolone cheese in favour of the presumed health benefits that lactose-free cheese could offer.

For the last cluster, comprised of young people holding a university degree, the national brand of the cheese, its organic production, and its PDO certification should be publicised. The quality guaranteed by these labels should be highlighted as well as the unique character and taste of the cheese due to the local environments; moreover, how production of such cheeses supports the local economies could be emphasised.

Given that the market share simulation showed that being a national brand helped to acquire the biggest market share, national producers, as well as modern distribution companies, should create effective strategies to take advantage of this preference.

Organic and EU quality certifications earned significant shares of the market; therefore, different messages that promote a traditional and environmentally friendly production method and the origin of cheeses should be used in promotion and advertising of these attributes. 

Although lactose-free cheese was not preferred by consumers, companies that nevertheless decide to use this label should concentrate their advertising strategies on the segment of lactose-intolerant people, communicating to them the possibility of enjoying the taste of dairy without the uncomfortable gastrointestinal symptoms that result from the ingestion of lactose.

Our study has some limitations. Investigating Provolone Valpadana cheese as the focal product prevents generalisation of the results to the entire category of quality-certified cheese, and thus, further research should be conducted on other cheeses. A more widely known cheese that could represent the entire category should be considered because evaluation of it would allow for inferences to all types of cheese. Moreover, a future pan-EU study could be conducted to reveal national market differences, which would allow for different strategies to be adopted in specific national and international contexts.

As for the methodology, we suggest the use of a questionnaire that would investigate not only consumer preference and knowledge of labels but also the relationships between these aspects; this could be followed by a sensory analysis (trained sensory panel) and integrated by an experimental auction where consumers would be asked to bid on the different cheese types and the top bidders on the binding product in the binding round would have to acquire the product.

Considering that exhortations to ‘buy national’ occur in times of economic turmoil in an attempt to protect domestic industry and jobs, in the future it would be interesting to study the role of protecting domestic industry and jobs through buying local products [40].

Finally, as consumers base their consumption decisions not only on economic reasons but also identities, culture, values, worldviews, and group memberships [41], the long-run benefits of purchasing domestic products and their support of the local economy [42] would be an area recommended for future study.

Finally, further studies should consider voluntary nutritional labels on FOP, named “Nutriscore” or “NutrInform battery “and other ones, on cheese products in combination with quality labelling in order to investigate the trade-off between these types of food labelling.

## Figures and Tables

**Table 1:** Attributes and their corresponding levels.

Attributes	Level
Price	2.20 € 2.86 € 3.20 €
European Quality Certification (Protected Designation of Origin)	Yes No
Production System	Organic Conventional
Lactose content	Contains Lactose Lactose-Free
Brand	Latteria Soresina Auricchio Coop No Brand

**Table 2:** Sociodemographic distribution of the collected sample in comparison with the Italian population (%).

Variables	Levels	Sample	Population
Age (in years)	18–35	24	
	36–55	53	
	>55	23	
Gender	Female	81	51 *
	Male	19	49 *
Education	Elementary and middle school	2	
	High school Diploma	41	
	University degree	57	
Marital status	Bachelor/Maiden	33	42 *
	Married	53	47 *
	Divorced	9	3 *
	In a relationship	3	
	Widow	1	
Family members	1	12	33 *
	2	36	27 *
	3–4	40	35 *
	>4	12	5 *
Geographical Distribution	North	27	46 *
	South and Islands	66	34 *
	Centre	7	20 *
Occupation	Office worker	40	
	Freelance	13	
	Student	5	
	Housewife	14	
	Teacher	2	
	Entrepreneur	2	
	Unemployed	5	
	Other	19	
Average annual income (€)	<10.000	26	
	10,000–20,000	31	
	20,000–40,000	32	
	40,000–50,000	4	
	>50,000	7	

* ISTAT (National Statistics Institute) data extracted in April 2020. http://dati.istat.it/Index.aspx?QueryId=18460.

**Table 3:** Mean part-worth and relative importance for all respondents.

Attributes	Levels	Utility Estimate	Importance Values (%)
Quality Certification	NO PDO	−0.077	8.292
	PDO	0.077	
Production System	Organic	0.144	10.652
	Conventional	−0.144	
Brand	No Brand	−0.287	58.239
	Aurrichio	0.966	
	Coop	−0.378	
	Latteria Soresina	−0.301	
Lactose Content	Contains Lactose	0.039	8.941
	Lactose-Free	−0.039	
Price	2.2	−0.068	13.877
	2.86	−0.136	
	3.2	−0.204	
(Constant)		6.723	

Notes: Pearson’s R = 0.992; Kendall’s tau = 1.000.

**Table 4:** A. Mean part-worth for the clusters. B. Relative importance for the clusters.

**A**				
**Attributes**	**Levels**	**Cluster 1 (44%)**	**Cluster 2** **(22%)**	**Cluster 3** **(34%)**
Price	2.20 €	−0.14947	−0.01865	−0.08053
	2.86 €	−0.1943	−0.02424	−0.10469
European Quality Certification (Protected Designation of Origin)	Yes	−0.0089	0.0658	0.2014
	No	0.0089	−0.0658	−0.2014
Production System	Organic	0.0656	0.0668	0.2971
	Conventional	−0.0656	−0.0668	−0.2971
Lactose content	Contains Lactose	0.0171	0.0708	0.0519
	Lactose-Free	−0.0171	−0.0708	−0.0519
Brand	Latteria Soresina	−0.3137	−0.8606	0.0878
	Auricchio	0.9577	2.7149	−0.1778
	Coop	−0.5533	−1.0091	0.2683
	No brand	−0.0900	−0.8440	−0.1800
**B**				
**Attributes**	**Relative Importance**			
	**Cluster 1**		**Cluster 2**	**Cluster 3**
Price	13.57		6.49	19.13
European Quality Certification (Protected Designation of Origin)	6.43		4.53	13.40
Production System	8.79		5.30	16.67
Lactose Content	8.38		6.52	11.23
Brand	62.83		77.16	39.57

**Table 5:** Sociodemographic characteristics of clusters.

Variables	Levels	Cluster 1	Cluster 2	Cluster 3
Age (in years)	18–35	20%	11%	39%
	36–55	61%	61%	36%
	>55	19%	28%	25%
Sig = 0.001 < 0.01 (1%)				
Education	Elementary and middle school	1%	8%	6%
	High school Diploma	49%	43%	24%
	University degree	51%	49%	70%
Sig = 0.003 < 0.01 (1%)				
Marital status	Bachelor/Maiden	27%	23%	48%
	Married	58%	4%	42%
	Divorced	10%	11%	7.5%
	In a relationship	5%	58%	2.5%
	Widow	0%	4%	0%
Sig = 0.020 < 0.05 (5%)				
Family members	1	14%	7%	15%
	2	18%	21%	32%
	3–4	56%	53%	6%
	>4	12%	19%	47%
Sig = 0.026 < 0.05 (5%)				
Geographical Distribution	North	23%	13%	36%
	South and Islands	70%	83%	53%
	Centre	7%	4%	11%
Sig = 0.010 < 0.05 (5%)				
Gender	Female	85%	91%	72%
	Male	15%	9%	28%
Sig = 0.010 < 0.05 (5%)				

**Table 6:** Simulation of market share.

Card	Maximum Utility	Bradley Terry-Luce	Logit	Quality Certification	Production System	Price	Quality Certification	Production System
1	29.8%	19.0%	27.1%	PDO	Organic	2.20	Aurrichio	Contains Lactose
2	19.9%	18.5%	23.4%	NO PDO	Organic	2.20	Aurrichio	Contains Lactose
3	12.6%	15.8%	13.2%	PDO	Organic	2.20	Coop	Contains Lactose
4	5.4%	15.1%	9.1%	PDO	Conventional	2.20	Coop	Contains Lactose
5	26.1%	17.2%	19.3%	NO PDO	Conventional	3.20	Aurrichio	Lactose-Free
6	6.2%	14.4%	7.8%	NO PDO	Conventional	3.20	Latteria Soresina	Contains Lactose
